# Teaching tracheal intubation: Airtraq is superior to Macintosh laryngoscope

**DOI:** 10.1186/1472-6920-14-144

**Published:** 2014-07-16

**Authors:** Hong Zhao, Yi Feng, Yanyan Zhou

**Affiliations:** 1 Department of Anaesthesiology and Pain Medicine, Peking University People’s Hospital, 11 Xizhimen South Street, Beijing, China

**Keywords:** Clinical education, Tracheal intubation, Evaluation of clinical performance

## Abstract

**Background:**

Tracheal intubation with Macintosh laryngoscope is taught to medical students as it is a lifesaving procedure. However, it is a difficult technique to learn and the consequences of intubation failure are potentially serious. The Airtraq optical laryngoscope is a relatively novel intubation device, which allows visualization of the glottic plane without alignment of the oral, pharyngeal, and tracheal axes, possessing advantages over Macintosh for novice personnel. We introduced a teaching mode featured with a progressive evaluation scheme for preparation and performance of tracheal intubation with medical students in this prospective randomized crossover trial who had no prior airway management experience to find the superior one.

**Methods:**

Twenty-six medical students of the 8-year programme in the 6th year participated in this trial, when they did their one-week rotation in the department of anaesthesiology. Each of the students intubated 6 patients, who were scheduled for surgeries under general anaesthesia, each laryngoscope for 3 patients respectively. One hundred and forty-nine consecutive patients scheduled for surgical procedures requiring tracheal intubation were enrolled. Patients were randomly allocated to undergo tracheal intubation using Macintosh (n = 75) or Airtraq (n =74) laryngoscope. The progressive evaluation scheme was applied to each intubation attempt.

**Results:**

Intubation success rate was significantly higher in Airtraq group than Macintosh group (87.8% vs. 66.7%, *P* < 0.05). Duration of glottis exposure was significantly shorter in Airtraq group compared to Macintosh group (50 ± 19 s vs. 81 ± 27 s, *P* < 0.001). A grade I Cormack and Lehane glottic view was obtained in 94.6% of patients in the Airtraq group versus 32% of patients in the Macintosh group (P <0.001). Duration of intubation in Airtraq group was significantly shorter (68 ± 21 s vs. 96 ± 22 s, P < 0.05) compared to Macintosh group.

**Conclusions:**

Airtraq laryngoscope is easier to master for novice personnel with a higher intubation success rate and shorter intubation duration compared with the Macintosh laryngoscope.

**Trial registration:**

Trial registration number is ChiCTR-TRC-13003987, approval date Dec 12, 2013.

## Background

Tracheal intubation is a lifesaving procedure, and an important step in cardiopulmonary resuscitation, which helps establishing an artificial airway. Therefore it is a basic technique to master for doctors in all fields. In operating theatre, tracheal intubation is usually applied after anaesthesia induction (loss of consciousness induced by medication) to facilitate a secured airway in cases involving neuromuscular paralysis and positive pressure ventilation. For medical students of 8-year programme in the 6th year, one-week rotation in the department of anaesthesiology is a good opportunity to learn this technique. Macintosh laryngoscope is the most widely used device to facilitate tracheal intubation [1], but is considered difficult to apply for novice personnel. It was reported that a 90% success rate requires 47 times of intubation practice [2]. Actually tracheal intubation could be divided into two main steps, glottis exposure and tube insertion. The main cause of difficulty with classic Macintosh blades and direct laryngoscopy lies in glottis exposure, which requires the alignment of oral, pharyngeal and tracheal axes [3].

**Figure 1:**
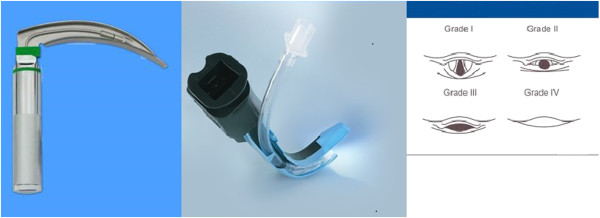
**Two laryngoscopes and Cormack-Lehane classification of glottis exposure.** Left is Macintosh laryngoscope, middle is Airtraq optical laryngoscope.

The Airtraq optical laryngoscope is a relatively new, single-use device for tracheal intubation. The curvature of the Airtraq blade and the special internal arrangement of the optical components allow visualization of the glottic plane without alignment of the oral, pharyngeal, and tracheal axes, which may facilitate an easier glottis exposure (Figure 1). Several studies revealed the advantages of Airtraq over Macintosh laryngoscopes in simulated intubation scenarios on manikins [4] and in clinical trials [5]. However, teaching medical students how to intubate with both devices has not been studied.

We here introduce our teaching mode for tracheal intubation featured with a progressive evaluation scheme for preparation and performance, and we compared the effect of teaching tracheal intubation with Macintosh and Airtraq laryngoscope to find the easier one to learn in a randomized crossover controlled clinical trial.

## Methods

### Study population and simulation-based medical education

This randomized crossover clinical trial was approved by Peking University People’s Hospital Institutional Review Board. All students and patients signed a written informed consent to participate in the study.

Twenty-six medical students of the 8-year programme in the 6th year participated in this trial, when they did their one-week rotation in the department of anesthesiology at Peking University People’s Hospital, a university hospital. They had no prior experience with either Macintosh or Airtraq laryngoscope (Prodol Meditec S.A., Vizcaya, Spain). Before their rotation, they were instructed airway evaluation and Cormack-Lehane classification (degree of glottis exposure) as well as airway management by lectures, written instructions, video presentations and line drawings of anatomical landmarks. On the first day of rotation, they would watch a video about all the above information. Subsequently, they would observe tracheal intubations with Macintosh and Airtraq laryngoscope on surgical patients by an attending anaesthesiologist, each laryngoscope on three patients respectively. All students performed 10 intubations on manikins (Laerdal® airway management trainer) with each laryngoscope. A progressive evaluation scheme for preparation and performance (Table 1) of tracheal intubation was adopted to emphasize the key points during intubations on manikins.

**Table 1:** Progressive evaluation scheme for tracheal intubation

	
1. Prepare the laryngoscope, anti-bite block, sticky tapes and stethoscope	10
For Macintosh laryngoscope, check the light, keep the light off during preparation, put a stylet in the tube and shape the tube For Airtraq laryngoscope, turn on the light, put the tube in the side channel	
2. Efficient mask ventilation	10
3. Proper extension of the atlantooccipital joint	10
4. Proper insertion of laryngoscope	10
Insert Macintosh laryngoscope from the right side of the mouth, move toward the midline	
Insert Airtraq laryngoscope along the midline	
5. Appropriate request for help to press cricoid or BURP (backward, upward, and right-sided pressure)	10
6. Efficient glottis exposure, Cormack-Lehane Grade I or II	10
7. Insert the tube into the trachea to an appropriate depth	10
8. Inflate cuff of the tube to an appropriate pressure	10
9. Auscultate both lungs to identify position of the tube, place anti-bite block, secure the tube with tapes	10
10. Time of intubation less than 150 s, calculated from opening the mouth to the first appearance of normal wave capnography	10
Inserted tube is too deep resulting in one-lung ventilation	-10
Failed intubation, i.e. tube not inserted into the tracheal within 150 s from opening the mouth or tube inserted into the oesophagus	-10

### Evaluation of clinical intubation

After the simulation-based medical education, each student performed six tracheal intubations using either Macintosh laryngoscope (n = 75) or Airtraq optical laryngoscope (n = 74) in this week of rotation under close supervision by an attending anaesthesiologist. We enrolled 149 consecutive American Society of Anaesthesiologists physical status I to II patients, aged between 18 and 65 years old, scheduled for surgical procedures requiring general anaesthesia and tracheal intubation. Exclusion criteria were a history or any indicator of a difficult airway (i.e. Mallampati grade >2, obesity (body mass index >30 m/kg^2^), interincisor distance less than 4 cm), or any risk factor of pulmonary aspiration. Patients were allocated into 2 groups, one group for Macintosh laryngoscope and the other for Airtraq laryngoscope, by a computer generated random number list and blinded to group assignment. After administration of oxygen, anaesthesia was induced with midazolum 0.03 mg/kg, propofol 2 mg/kg, rocuronium 0.6 mg/kg and fentanyl 3 μg/kg. Mask ventilation was performed for 2 minutes using 100% oxygen. Afterwards tracheal intubation was performed by one student, using Macintosh or Airtraq laryngoscope according to the randomization sequence. Endotracheal tubes of inner diameter (ID) size 8.0 mm were used for male adult patients, and size ID 7.5 mm for female patients. An intubation stylet was used for intubation with Macintosh. Optimization maneuvers were applied to improve the glottic view when required. The progressive evaluation scheme was also applied to assess student’s performance for each intubation attempt (Table 1).

Duration of glottis exposure was defined as the period from opening the mouth to the maneuver to insert the endotracheal tube. Duration of successful intubation was defined as the period from opening the mouth to the first appearance of normal wave for capnography. Intubation failure was defined as when it was not completed within 150 seconds or it resulted in an oesophageal intubation. In case of a failure, the intubation would be accomplished by an attending anaesthesiologist as soon as possible giving priority to oxygenation. To ensure patients’ safety, only one attempt was allowed by the student to intubate the patient with either laryngoscope, and the whole process was under close supervision of an attending anaesthesiologist. Primary outcome was the success rate of intubation using each laryngoscope. The number of optimization maneuvers required to perform tracheal intubation was also recorded. Optimization maneuvers, performed to improve the line of sight, should include, attention for positioning, head tilt, BURP (backward, upward, and right-sided pressure) and slight movements of the blade once placed into the vallecula in an attempt to lift the epiglottis. Dental trauma, visible trauma to lip or oral mucosa, and presence of blood on laryngoscope blade would be recorded. At the end of rotation, each student rated the difficulty of learning each technique on a number rating scale about, ranging from 1-5, with 1 = extremely easy, 5 = extremely difficult.

### Statistical analysis

An approximate success rate for tracheal intubation for novice intubators ranges from 35% to 65% [6,7]. The sample size was calculated based on an alpha error of 0.05 and a beta error of 0.2, with a minimal difference of 20% in terms of the intubation success rate (compared to 65%). The resulting minimal number of patients to be intubated was 71 per group. Since each medical student performed three intubations, 24 medical students were needed for each group.

Continuous data are presented as means ± SD and ordinal and categorical data are presented as numbers and frequencies, respectively. Comparisons between groups were analyzed with independent *t* test, Mann-Whitney U test or χ ^2^ test. *P* < 0.05 was considered statistically significant.

## Results

Due to limited time of rotation, 7 out of 26 students only intubated 5 patients, 19 students intubated 6 patients (Table 2) and a total of 149 consecutive patients underwent this randomized crossover study. There were no differences in demographic data or baseline airway variables between the groups (Table 3). Intubation success rate in Airtraq group was 87.8%, which was significantly higher than in the Macintosh group (66.7%, *P* < 0.05) (Table 4). Duration of glottis exposure was significantly shorter in Airtraq group compared to Macintosh group (50 ± 19 s vs. 81 ± 27 s, *P* < 0.001). A grade I Cormack and Lehane glottic view was obtained in 94.6% of patients in the Airtraq group, but only in 32% of patients in the Macintosh group (P <0.001). No optimization maneuvers were required to improve the glottis exposure of patients in the Airtraq group, while 33.7% of patients in the Macintosh group required adjusting the view (P < 0.001). Duration of intubation in Airtraq group was significantly shorter compared to Macintosh group (68 ± 21 s vs. 96 ± 22 s, *P* < 0.05). Finally, medical students rated Airtraq easier to learn than Macintosh laryngoscope (Intubation Difficulty number rating scale being 2.2 ± 0.7 vs. 2.8 ± 0.6, *P* < 0.05). No dental, lip, mucosa trauma or other complications were identified in either study group. For the failed cases, intubations were accomplished by an attending anaesthesiologist as soon as possible giving priority to oxygenation. The Cormack-Lahane classification was all rated grade I by the experienced attending anaesthesiologists for those failed cases and all failed intubations intubated with one single attempt by the attending physician.

**Table 2:** Successful intubation for each intubation attempt

	**Macintosh (n of success/total)**	**Airtraq (n of success/total)**
First intubation	9/26	19/26
Second intubation	23/26	24/26
Third intubation	18/23	22/22

**Table 3:** Demographic Data for Patients

**Group**	**Macintosh (n = 75)**	**Airtraq (n = 74)**
Age,yr	49 ± 17	48 ± 18
Gender (M/F), n	27/48	33/41
Height (cm)	165.0 ± 5.8	164.9 ± 7.6
Weight (kg)	60.8 ± 8.1	63.8 ± 8.2
ASA classification (I/II), n	48/27	42/32
Mallampati Grade (I/II), n	56/19	52/22

Data are given as Mean ± SD.

**Table 4:** Intubation data for novice personnel using Macintosh and Airtraq Laryngoscope

**Group**	**Macintosh (n = 75)**	**Airtraq (n = 74)**
Overall success rate (%)	66.7%	87.8%§
Success rate first attempt (%)	34.6%	73.1%§
second attempt (%)	88.5%	92.3%
third attempt (%)	78.3%	100%§
Duration for glottis Exposure (s)	81 ± 27	50 ± 19*
Grade 1 for glottis exposure (n (%))	24 (32%)	70 (94.6%)*
Grade 2 for glottis exposure (n (%))	46 (61.3%)	4 (5.4%)*
Requirement for optimization maneuver (n (%))	25 (33.7%)	0*
Duration for Intubation (s)	96 ± 22	68 ± 21*
Intubation Difficulty NRS cu1-5)	2.8 ± 0.6	2.2 ± 0.7^§^

Data are given as Mean ± SD.

*P < 0.001 versus Macintosh group patients.

^§^P < 0.05 versus Macintosh group patients.

## Discussion

Our study demonstrated that Airtraq is a superior device for novice medical students to acquire tracheal intubation skills, with a higher success rate, less duration of intubation, better glottis exposure and less optimization maneuvers required.

Several investigators have reported that the mean success rate for the first 10 intubations via direct laryngoscopy by medical personnel untrained in tracheal intubation is about 35–65% and that an average of 47 attempts are needed to achieve a 90% success rate of intubation [2]. A more recent study showed a success rate of 20.6% for medical students [8], which was lower than the result of our study (Macintosh group 66.7%). In our study, the students achieved a relatively comparable high success rate with only one attempt allowed in contrast to 3 attempts allowed in other novice studies [9-11]. We considered the greatly improved performance was due to the simulation-based learning and the progressive evaluation scheme applied to every intubation attempt, which helped the students to familiarize with the upper airway anatomy and bear in mind the key points of intubation. For medical students, the difficulty of tracheal intubation lies in the diverse patient intubation conditions and the psychological burden fearing inducing complications. Simulation-based learning helped them to master the technique, get skillful on manikins and improve confidence to practice on real patients. According to our knowledge, it was the first time the progressive evaluation scheme was introduced to teaching medical students how to intubate, which helped the student to understand and memorize the key points and steps of intubation technique. The progressive evaluation scheme was adapted from the criteria for tracheal intubation trainee [2], in combination with our clinical and teaching experience, which has been applied in our university hospital for several years to teach novice personnel to learn this difficult but important technique.

Tracheal intubation using Macintosh laryngoscope has a high failure rate when performed by untrained medical personnel [7,8]. The main cause of difficulty lies in the alignment of oral, pharyngeal and tracheal axes to expose the glottis with Macintosh. While the alignment is not necessary with Airtraq due to the blade curvature and the special internal arrangement of the optical components, i.e. indirect laryngoscopy to allow visualization of the glottic plane. That was why the students could facilitate a quicker and better glottis exposure with Airtraq in our study, thus a shorter duration of intubation and higher success consequently. The findings suggested that Airtraq is easy to learn, which was consistent with the results of a recent meta-analysis [12]. Although the first three intubations in a person’s lifetime could be considered equally new experiences, we still witnessed the gradual increase of intubation success rate from the first intubation to the third one. But the success rate of the third intubation with Macintosh was decreased compared with the second one, which means the difficulty in learning this technique.

Our study was limited by the one-week rotation, which was too short to allow all students to perform six intubations, 3 intubations with each laryngoscope. Seven out of 26 students performed five intubations eventually. Although each intubation was treated as an independent performance, we could not rule out inter-individual variability in learning.

## Conclusions

Intubation failure is really dangerous for patients, sometimes could be fatal. Tracheal intubation with Airtraq compared with Macintosh is an easier way to learn and achieve success, which could help the students to build up confidence about learning and mastering this life-saving technique, and further to benefit the patients.

## Competing interests

The authors declare that they have no competing interests.

## Authors’ contribution

HZ helped design the study, collect data and write the manuscript. YF, the corresponding author, helped design the study. YYZ helped collect data. All authors read and approved the final manuscript.

## Pre-publication history

The pre-publication history for this paper can be accessed here:

http://www.biomedcentral.com/1472-6920/14/144/prepub
